# Two sustainable chromatographic approaches for estimation of new combination of phenylephrine hydrochloride and doxylamine succinate in presence of doxylamine oxidative degradation product

**DOI:** 10.1038/s41598-025-22295-6

**Published:** 2025-10-23

**Authors:** Heidi R. Abd El-Hadi

**Affiliations:** Faculty of Pharmacy, Pharmaceutical Chemistry Department, Egyptian Russian University, Badr City, Cairo, Egypt

**Keywords:** Anti allergic mixture, HPLC, HPTLC, Stability, Sustainability, Analytical chemistry, Green chemistry

## Abstract

A new drug combination of phenylephrine hydrochloride (PHE) and doxylamine succinate (DOX) has been introduced for treating allergic rhinitis. Stability testing is critical for uncovering degradation routes and assessing the stability of combined drugs. This study illustrates the application of two eco-friendly chromatographic techniques which are reversed phase high-performance liquid chromatography (RP-HPLC) and high-performance thin-layer chromatography (HPTLC), for assessing PHE and DOX when DOX oxidative degradation product (DOX DEG) is present. Using liquid chromatography- mass spectroscopy to identify DOX DEG. The HPLC method produced the best separation with isocratic elution and a mobile phase consists of ethanol and 0.01 M phosphate buffer pH = 5.0 (30: 70, v/v), and it was pumped at 1.0 mL/min. The analytes were measured at 260.0 nm using diode array detector (DAD), and the Xterra C_18_ column (100 mm × 4.6 mm × 5 m) used as a stationary phase. The method demonstrated a linear response for DOX and PHE across a concentration range of 5.00 to 100.00 µg/mL. The range for DOX DEG was 5.00 to 30.00 µg/mL. The limits of detection (LOD) were determined to be 1.44 for DOX, 1.59 for PHE, and 0.84 µg/mL for DOX DEG. Correspondingly, the limits of quantification (LOQ) were 4.32, 4.77, and 2.52 µg/mL for DOX, PHE, and DOX DEG, respectively. The separation in HPTLC was accomplished by combining ethanol, methylene chloride, and ammonia 30% in ratio 7:2.5:0.5 (v/v/v) as a developing system. The drugs were then quantitatively determined at wavelengths of 260.0 nm using UV detector. The linearity range was 4.00–26.00 (µg/band) for DOX and PHE while it was 0.50–10.00 (µg/band) for DOX DEG. Values of LOD were 0.65 ,0.76 and 0.16 µg/band for PHE, DOX, DOX DEG, respectively. While1.95,2.28 and 0.48 µg/band were values of LOQ. Per ICH guidelines, two analytical methods were validated and proven to be reliable, reproducible, and selective. Additionally, sustainability assessments confirmed their green credentials and practical applicability.

**Supplementary Information:**

The online version contains supplementary material available at 10.1038/s41598-025-22295-6.

## Introduction

 Green Analytical Chemistry aims to develop environmentally friendly methods for routine pharmaceutical testing, which is a major concern for quality control analysts^1^. The Analytical Procedure Index (GAPI) and the Analytical GREEnness metric (AGREE) are tools for evaluating the environmental impact, or “greenness,” of analytical techniques. Additionally, the Blue Applicability Grade Index (BAGI) offers a quantitative way to assess the applicability and practicality or “blueness” of these methods^2^.

**Fig. 1:**
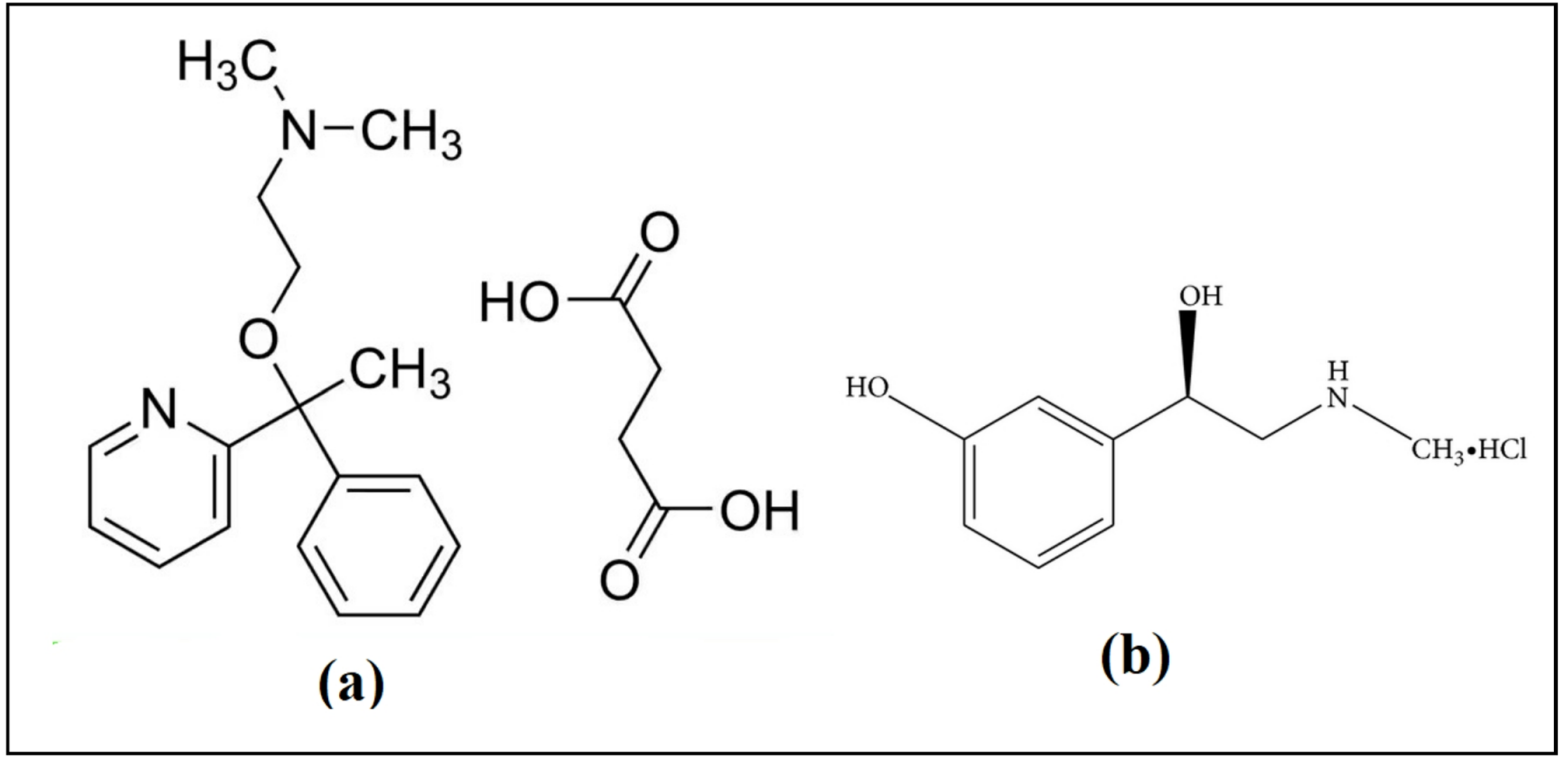
Chemical structures of doxylamine succinate (**a**) and phenylephrine hydrochloride (**b**).

Phenylephrine hydrochloride (PHE) with the chemical name (1R)−1-(3-hydroxyphenyl)−2-(methylamino) ethanol hydrochloride, is a sympathomimetic drug and it is used as a decongestant (Fig. 1)^3^. Doxylamine succinate (DOX) is an antihistamine with sedative properties. The chemical name is dimethyl ({2-[1-phenyl-1-(pyridin-2-yl)ethoxy]ethyl}) amine (Fig. 1)^4^. The trade name Poly Hist Forte^®^ tablets refer to a combination of PHE and DOX (Fig. 1). This formulation is widely used to treat runny or stuffy nose, sneezing, itching, watery eyes, and sinus congestion caused by allergies, colds, or the flu.

To enhance the stability of the active pharmaceutical ingredient and its formulations, there is an increasing demand for the isolation, detection, measurement, and characterization of both likely and potential degradation products^5^. To determine the drug’s intrinsic stability profile, ICH guidelines on forced degradation require stress testing. The most suitable stability-indicating method is one that can precisely measure and separate the drug’s degradation products^4^.

A review of the literature reveals that DOX has been quantified individually by FTIR spectroscopy^6^ and in the presence of its alkaline degradant using HPLC^7^. Methods also exist for the determination of DOX in multi-component formulations with other drugs using HPLC^8,9^ and chemometric techniques^10,11^. PHE was determined by spectrophotometric and HPLC approaches^12,13^. DOX and PHE were determined before in presence of Dextromethorphan HBr (DEX) as a ternary mixture by HPLC method^9^.However, based on current knowledge, no green and time saving research has been done to simultaneously determine DOX and PHE together in their binary dosage form, or when mixed with any of their degradation products.

The objective of this research is to develop two sustainable and selective chromatographic techniques (HPLC and HPTLC) for determining PHE and DOX in binary dosage form and when DOX oxidative degradation (DOX DEG) is present for the first time. With the aid of liquid chromatography mass spectrometry (LC-MS), the degradation product was described and clarified.

## Experimental

### Apparatus

#### HPLC method

The Waters 2695 LC separation module, Waters 996 DAD (200–600 nm) version 2, pump with low-pressure mixing system, and vacuum degasser were the LC systems utilized. Auto-sampler with a 120 vial capacity and a 100 µL sample loop. Empower software was used to process and monitor the output signal (https://www.waters.com/nextgen/us/en/products/informatics-and-software/chromatography-software/empower-software-solutions.html). The AJENWAY 3510 pH meter from Staffordshire (England) with glass combination was used to measure pH. The column was an Xterra C_18_ column (100 mm × 4.6 mm × 5 μm), USA.

#### HPTLC method

HPTLC densitometer (CAMAG, Switzerland, Muttenz) A Model 3 densitometer CAMAG TLC Scanner 3 version 3 with winCATS software. Precoated TLC sheets, silica gel 60 F254 (20 cm × 20 cm) plates (Merck), and a Linomat 5 autosampler with a 100 µL CAMAG micro-syringe are all included.

#### DOX degradation

A XEVO TQD triple quadruple instrument was used to perform ESI-MS negative ion acquisition mode. Waters Corporation mass spectrometer, Milford, MA01757, USA.

### Chemical and reagent

#### Pure sample

Mash Premiere for Pharmaceutical Industry (Cairo, Egypt) and Orchidia Co. for Pharmaceutical Ind. (Cairo, Egypt) kindly donated the DOX and PHE used in this experiment, respectively. According to the official methods, the purities of PHE and DOX were 98.52 ± 0.48% and 99.07 ± 0.72%, respectively^14^.

### Solvents

#### HPLC method

Ethanol HPLC grade from Merck, Darmstadt, Germany was utilized. The lab produced LC-grade water, which was then filtered using 0.45 μm membrane filters. 1.36 g of potassium dihydrogen phosphate (Oxford, India) was dissolved in 1000 mL of volumetric flask, and the volume was filled with LC-grade water. The pH was then adjusted using either phosphoric acid or sodium hydroxide to create phosphate buffer (pH 5.0; 0.01 M) (Biotech, Egypt)^14^.

#### HPTLC method

Every chemical and solvent used was of analytical grade and didn’t require any additional purification. Methylene chloride, 30% ammonia, and ethanol (Merck, Darmstadt, Germany) were utilized.

### Pharmaceutical formulation

Poly Hist Forte^®^ tablets were manufactured by Poly Pharmaceuticals, Inc (USA). Each tablet claimed to contain 10.5 mg for each of DOX and 10.0 mg of PHE as active ingredients.

### DOX oxidative degradation (DOX DEG) preparation

To prepare the degraded DOX solution, 100 mg of DOX was refluxed in ethanol with 30% H₂O₂ for 7 h. The mixture was evaporated to dryness, and the residue was dissolved and diluted to 100 mL with ethanol to create a 1.0 mg/mL stock solution. This stock was subsequently diluted with mobile phase in HPLC method and with ethanol in HPTLC method to a final working concentration of 100.0 µg/mL^8^.

### Standard solution

#### HPLC method

PHE, DOX, and DOX DEG stock standard solutions (1.0 mg/mL) were made independently by adding 50.0 mg of each one to a 50 mL volumetric flask, dissolving it in a small amount of ethanol, and then topping it off with a mobile phase. To create working standard solutions (100.0 µg/mL), 1 mL of each drug’s stock standard solution was transferred into a 100 mL volumetric flask and the volume was then finished using the same solvent.

#### HPTLC method

Using ethanol as a solvent, separate stock standard solutions of DOX, PHE, and DOX DEG (1.0 mg/mL) were made. The working standard solutions (100.0 µg/mL) were diluted with ethanol.

### Chromatographic condition

#### HPLC method

A mobile phase consisting of ethanol and 0.01 M phosphate buffer (pH 5.0) in ratio 30:70 v/v allowed for the best possible separation. After passing through a 0.45 μm membrane, the mixture was ultrasonically degassed for half an hour. An Xterra C_18_ column (100 mm × 4.6 mm, 5 μm particle size) was used for an isocratic elution, which was run for 10 min at a flow rate of 1 mL/min. The column was conditioned with the mobile phase for thirty-five minutes before the sample was injected. After injecting a 20 µL sample, a diode array detector (DAD) set at 260.0 nm was used for detection. The procedure was carried out at room temperature.

#### HPTLC method

The stationary phase for the chromatographic separation was 10 cm × 20 cm TLC aluminum sheets coated with silica gel 60 F254 (Merck). Ethanol, methylene chloride, and ammonia 30% were combined in ratio 7:2.5:0.5 (v/v/v) to form the mobile phase. Using a scanning rate of 20 mm/s, solutions of the examined medications were applied as discrete, compact spots 15 mm from the plate’s bottom edge. Each spot had a 6 mm band width and a slide size of 6.0 mm × 0.3 mm. The TLC plates were activated by heating them to 100 °C for 20 min before use to remove any remaining moisture^15^. Before development, the chromatographic tank was saturated with each mobile phase separately for 30 min. After being developed over 8 cm in an ascending fashion and allowed to air dry, the normal-phase TLC plates were specifically scanned at 260.0 nm.

## Method validation

Validation of the developed techniques was carried out as stated by ICH guidelines^16^.

### Linearity

#### HPLC method

In three distinct sets of 10-mL volumetric flasks, aliquots corresponding to 5.00–100.00 µg/mL for both PHE and DOX and 5.00–30.00 µg/mL for DOX DEG were precisely and independently transferred from their working standard solution. The volume was then filled to the appropriate level using the mobile phase. 20 µL of each concentration was injected into the analytical column, and separation was accomplished in accordance with the “Chromatographic Conditions” section. The regression equations were then calculated after a linear calibration curve was obtained for each of PHE and DOX and DOX DEG relating ratio peak area (using 60.00 µg/mL of each of PHE, DOX and 20.00 µg/mL of DOX DEG).

#### HPTLC method

PHE and DOX concentration ranges were 4.00–26.00 µg/band, while DOX DEG ranged from 0.50 to 10.00 µg/band. Calibration curves were created by plotting concentrations against relative peak area ratios, using 14.00 µg/band of DOX and PHE and 6.00 µg/band of DOX DEG as reference standard. These plots were then used to calculate regression equations.

### Accuracy

#### HPLC method

The recommended HPLC method was used for estimating different concentrations of DOX, PHE, and DOX DEG (30.00, 70.00, 90.00 µg/mL for DOX and PHE, and 12.00, 18.00, 22.00 µg/mL for DOX DEG) in triplicate to ensure accuracy. The concentrations were then calculated using the regression equations for each drug.

#### HPTLC method

To guarantee the accuracy of the results, the suggested HPTLC method was used to measure the various concentrations of DOX, PHE, and DOX DEG (5.00, 15.00, and 20.00 µg/band for DOX and PHE, and 3.00, 5.00, and 9.00 µg/band for PHE) in triplicate. The regression equations for each drug were then used to calculate concentrations.

### Precision

The same concentrations previously mentioned in accuracy were examined in triplicates intra-daily and over the course of three consecutive days using the previously described procedures under linearity for HPLC and HPTLC. After that, RSD% was determined for every sample.

### Specificity

Rt, Rf values, and resolution were acquired to evaluate the separation of the investigated compounds using the methods that were described and to demonstrate the specificity of the suggested methods.

### Robustness and system suitability parameters

By examining the impact of specific parameters, such as the mobile phase flow rate, percentage of mobile phase components, and detection wavelength in HPLC, and the percentage of mobile phase components and saturation time in HPTLC, robustness was determined. Capacity factors, tailing factors, column efficiency, selectivity, and resolution factors for both HPLC and HPTLC were calculated to guarantee the system suitability parameters.

### Application to pharmaceutical formulation and statistical analysis study

In a clean mortar, ten Poly Hist Forte^®^ tablets were precisely weighed, crushed, and thoroughly combined. A measured amount of tablet powder was added to a 25 mL volumetric flask that had 15 mL of ethanol in it. After 30 min of sonicating the mixture, ethanol was used to bring the volume to the desired level. Whatman filter paper with a pore size of 0.5 μm was used to filter the final solution. To achieve concentration within the defined linearity ranges for both DOX and PHE, appropriate dilutions were subsequently made using the same solvent. The suggested techniques described in the linearity section were used to analyze the dosage form solutions, and the computed regression equations were used to ascertain the analyte concentrations. The results of the applied methods were compared to those of the official approach using the Student’s t-test, F-test^14^.

### Greenness assessment

Green analytical chemistry focuses on making analytical procedures more environmentally benign and safer to humans. Greenness assessment is the methodical analysis of a procedure, product, or approach to ascertain its overall sustainability and environmental impact. The environmental impact of analytical techniques was assessed using four key criteria: high energy consumption, significant waste generation, extensive chemical consumption with associated hazards, and the use of large volumes of reagents^17^. Two greenness evaluation tools were used to assess the proposed method’s cost-effectiveness and eco-friendliness simultaneously which are:

#### Analytical greenness metric (AGREE)

The accompanying software (https://mostwiedzy.pl/AGREE) provides both qualitative and quantitative assessments of analytical methods’ greenness. It is a simple, all-inclusive tool to evaluate the environmental friendliness of analytical methods using the 12 principles of green analytical chemistry as evaluation criteria. A clock-like diagram visually displays the evaluation results, with a color scale representing the level of greenness. These criteria include treatment, sample amount and stages, waste, energy consumption, and toxicity. Each of these variables is converted into a single 0–1 scale^18^. The width of the segment to which each principle is related, and the color scale of the resulting pictogram serve as indicators of the process and weight of each principle^11^. With values near 1 and a dark-green background, the evaluated methods are shown to be more environmentally friendly in the pictogram’s center^19^.

#### Green analytical procedure index (GAPI)

GAPI is a novel assessment tool that builds upon the foundation of the Analytical Eco-Scale to provide a thorough, qualitative evaluation of a method’s environmental impact across its entire lifecycle, from initial sampling to final analysis^20^ (https://mostwiedzy.pl/en/justyna-plotka-wasylka,647762-1/gapi). Five pentagrams, each of which represents a step in the analytical process, are used in this tool to provide comprehensive information on fifteen different aspects of the analytical technique^20^. These include the following: the analytical method’s overall goal, the solvents and reagents utilized, instrumentation, sampling, and preparation^21^. The GAPI color scheme states that red denotes a significant environmental risk, yellow denotes less ecological tolerance, and green denotes greater ecological tolerance^22^.

### Blue applicability grade index (BAGI)

BAGI is a new metric for assessing the functionality of an analytical procedure in practical settings. It complements green metrics by focusing on practical aspects of White Analytical Chemistry^23^. BAGI was calculated by the following software (bagi-index.anvil.app). The analytical chemistry method’s practicability is measured using a scale from 25 to 100; the higher the score, the more useful the method^23^. The type of analysis, the number of analytes that are determined simultaneously, the number of samples that can be analyzed in an hour, and the kind of reagents and materials used in the analytical method are the ten main characteristics that this tool evaluates^24^. The amount of samples, the type of sample preparation, the degree of automation, the number of samples that can be treated concurrently, the instrumentation required, and the necessity of preconcentration are all included^25^.

## Result and discussion

Stability assessment aims to demonstrate how environmental factors such as temperature, humidity, and light affect the quality of a drug or product over time. This procedure helps to define a re-test period, ensuring that the drug remains stable and effective. Stability-indicating methods are critical for identifying degradation, ensuring safety, and serving as quality control tools^26^.

Degradation products are undesirable compounds that can form during manufacturing, transportation, or storage because of environmental factors such as light, heat, moisture, or reactions with excipients and packaging^27^. As a result, monitoring the degradation of products is an important aspect of drug development. To the best of our knowledge, no previous research has focused on separating the two compounds either individually or in the presence of one of their degradation products. In this study, a stability investigation was carried out with the goal of completely separating and identifying the degradation product within the drug combination being evaluated. This emphasizes the importance of our work, which introduces first, fast, reliable and time-saving RP-HPLC and HPTLC techniques.

### Degradation behavior of DOX

According to the proposed degradation pathway, an ether group was changed into an alcoholic one to produce an alcohol derivative. Furthermore, a nitrogen atom oxidation was observed in the pyridine ring as indicated in (Fig.S1)^8^. Using LC-Mass, the degradation product was clarified (Fig. S2)^8^.

### Method development and optimization

#### HPLC method

**Fig. 2:**
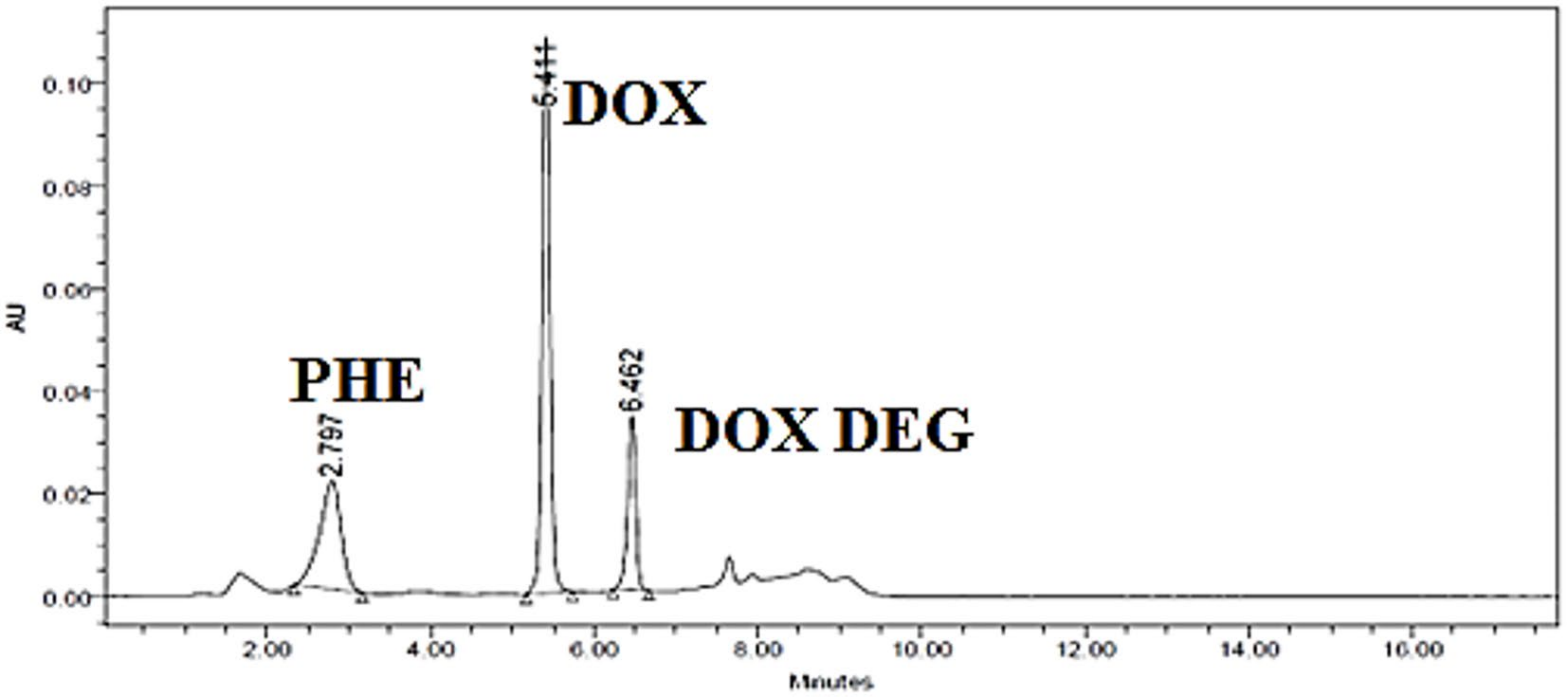
HPLC chromatogram of phenylephrine hydrochloride (Rt: 2.797 min), Doxylamine succinate (DOX) (Rt: 5.411 min) and Doxylamine succinate degradation (DOX DEG) (Rt: 6.462 min) using mobile phase composed of ethanol and 0.01 M phosphate buffer pH = 5.0 (30: 70, v/v), with flow rate 1 mL/min.

Several solvent systems with varying ratios, pH levels, and flow rates were tested, including methanol: water (30:70 and 70:30, v/v) and acetonitrile: methanol (60:40, v/v). The effects of various mobile phase pH values, including 4.0, 5.0, and 6.0, on the analytes’ peak resolution and retention durations were examined. It was found that the drugs under study eluted rapidly at pH values below 5.0, while the peaks of the analytes under study appeared unacceptable late at pH values above 5.0. We investigated the effects of flow rate (0.8–1.2 mL/min) on the analyte peak retention times. 1 mL/min was the ideal flow rate for efficient drug separation in a reasonable amount of time. The optimal separation and peak symmetry for DOX, PHE, and DOX DEG was obtained using ethanol and 0.01 M potassium dihydrogen phosphate (pH 5.0) in a 30:70 (v/v) ratio on an Xterra C_18_ column (100 mm × 4.6 mm, 5 μm) at a flow rate of 1.00 mL/min. The detection was done with a DAD at 260.0 nm. All three compounds were successfully separated in 10 min, with retention times of 2.79 min for PHE, 5.41 min for DOX, and 6.45 min for DOX DEG (Fig. 2).

#### HPTLC method

**Fig. 3:**
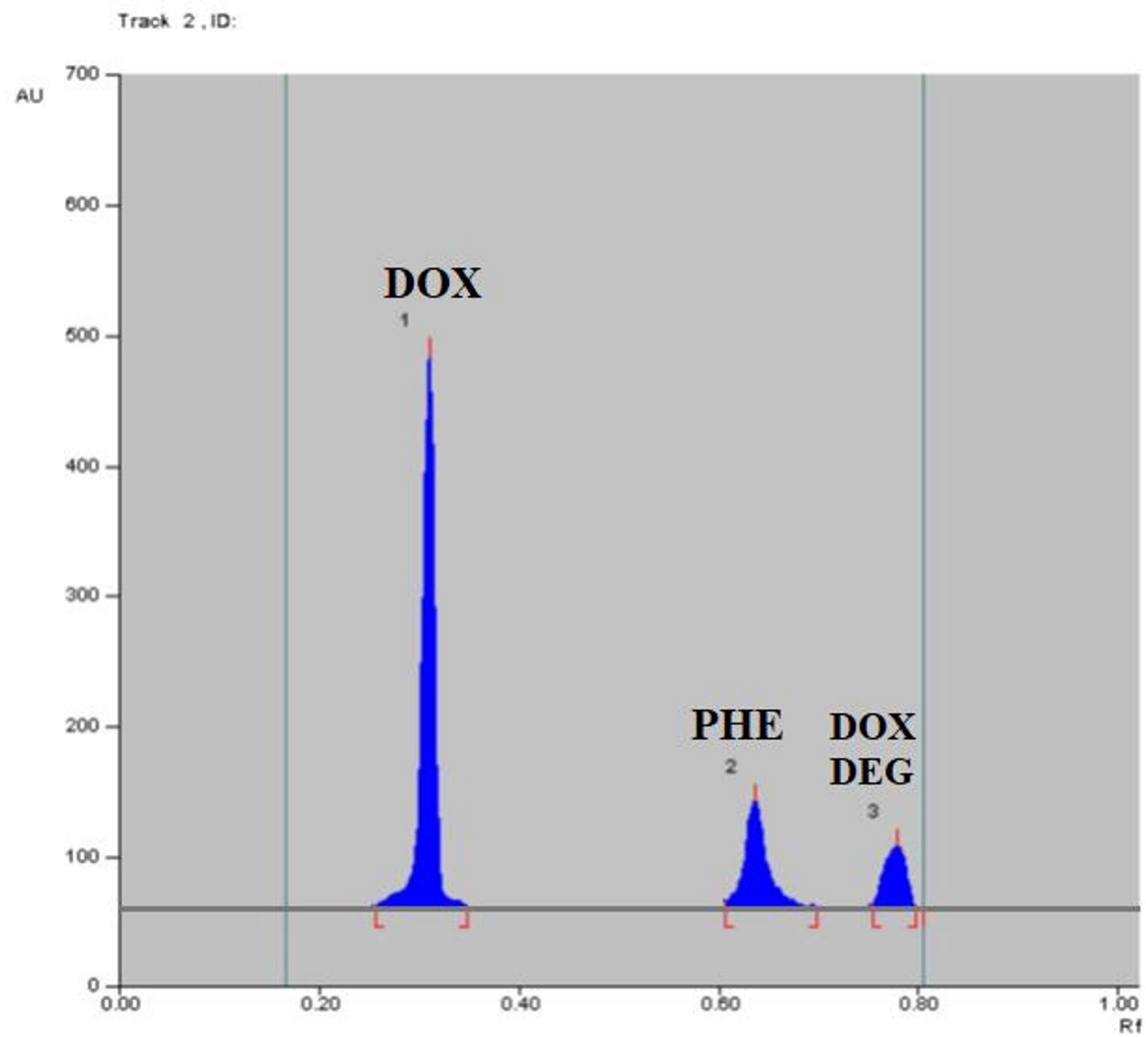
2D HPTLC chromatogram of mixture of DOX, PHE and DOX DEG with the following RF 0.31 ± 0.02, 0.64 ± 0.02 and 0.78 ± 0.02, respectively.

**Fig. 4:**
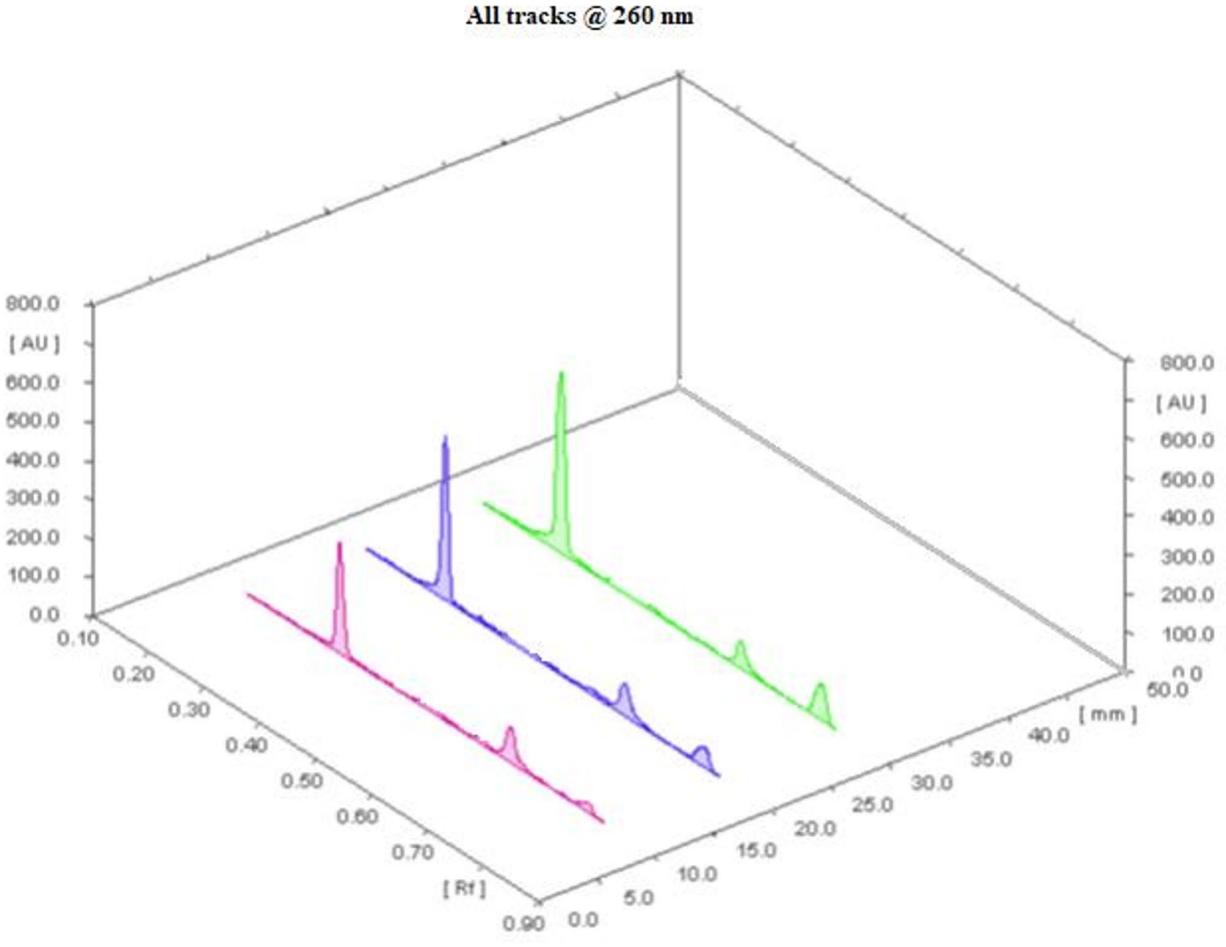
3D HPTLC chromatogram of laboratory prepared mixtures of DOX, PHE and DOX DEG at 260.0 nm.

The most time-consuming step in developing the HPTLC method is typically determining the best solvent system. Non-polar solvents such as chloroform, benzene, and toluene are commonly used in development systems, but we exclude them from our trials due to their known environmental toxicity^15^. Experiments with different mobile phase systems, including butanol, ethyl acetate, and ammonia, did not yield satisfactory separation results. Other systems, including butanol, ethyl acetate, and acetic acid, did not improve separation. PHE, DOX, and DOX DEG were separated using ethanol: methylene chloride: ammonia 30% (7.0:2.5:0.5, v/v/v) and HPTLC at 260.0 nm. The RF values for DOX, PHE, and DOX DEG were 0.31 ± 0.02, 0.64 ± 0.02, and 0.78 ± 0.02, respectively (Figs. 3 and 4). Wavelengths (220.0, 240.0, 260.0, and 310.0 nm) were tested for densitometric measurements, and it was determined that 260.0 nm was the most sensitive for all analytes.

### Method validation

#### Linearity and range

After plotting calibration graphs that connected the peak area ratios to the corresponding PHE, DOX and DOX DEG concentrations, the corresponding regression equations were computed to guarantee linearity, as indicated in (Table 1).

**Table 1:** Regression and validation parameters of the proposed chromatographic methods for determination of PHE, DOX and DOX DEG ^a^ average of three different concentrations repeated three times within the day. ^b^ precision was evaluated by measuring the response of three concentrations of each drug three separate times on the same day (repeatability) and on three different days (intermediate) precision. ^c^ LOD and LOQ were calculated from the standard deviation (s) of the response and the slope of the calibration curve (S) according to the following equations: LOD = 3.3(s/S) and LOQ = 10(s/S).

Method parameters	HPLC method			HPTLC method		
	PHE	DOX	DOX DEG	PHE	DOX	DOX DEG
Calibration range	5.00–100.00 (µg/mL)		5.00–30.00 (µg/mL)	4.00–26.00 (µg/band)		0.50–10.00 (µg/band)
Linearity						
Correlation coefficient (*r*)	0.9998	0.9998	0.9996	0.99962	0.9994	0.9998
Slope	0.01695	0.01254	0.058203	0.085106	0.063266	0.166697
Intercept	0.000835	0.001236	−0.18098	−0.174	0.111003	0.005729
Accuracy ^a^ (Mean% ±SD)	100.26 ± 0.87	100.29 ± 0.77	100.00 ± 1.23	100.34 ± 0.887	100.92 ± 0.79	100.15 ± 0.97
Precision ^b^(RSD %)						
Repeatability	0.84	1.00	1.01	0.93	0.60	1.15
Intermediate	0.78	0.82	0.63	0.80	0.36	0.99
LOD ^c^	1.59 (µg/mL)	1.44 (µg/mL)	0.84 (µg/mL)	0.65 (µg/band)	0.76 (µg/band)	0.16 (µg/band)
LOQ ^c^	4.77 (µg/mL)	4.32 (µg/mL)	2.52 (µg/mL)	1.95 (µg/band)	2.28 (µg/band)	0.48 (µg/band)

#### Accuracy

By examining different concentrations, the accuracy of the proposed methods was evaluated. The recovery percentage and standard deviation were then calculated, as indicated in (Table 1).

### Precision

Table 1 presents the findings of PHE, DOX and DOX DEG’s repeatability and intermediate precision. The repeatability and intermediate precision RSD% were less than 2%, demonstrating the reproducibility and dependability of the assay’s methodology.

### Specificity

As a separation approaches, the method’s good selectivity was determined by calculating system suitability parameters, particularly resolution.

### System suitability testing and robustness

Table 2 shows that the system suitability parameters for the suggested HPLC and HPTLC methods were calculated and that FDA recommendations were successfully met. According to the results in (Table 3), the suggested approaches are reliable and unaffected by minor, intentional changes.

**Table 2:** System suitability parameters for PHE, DOX and DOX DEG in the proposed methods.

Parameters	HPLC method			HPTLC method			Reference value
	PHE	DOX	DOX DEG	DOX	PHE	DOX DEG	
Retention time (Rt)	2.79 min	5.41 min	6.45 min	-			-
Retardation factor (Rf)	-			0.31	0.64	0.78	-
Resolution (Rs)	-	7.68	6.03	-	7.42	3.16	> 1.5
Capacity factor (K)	1.79	4.41	5.46	2.22	0.56	0.28	0–10
Tailing factor (T)	0.95	1.02	0.96	0.95	1.17	1.07	≤ 2
Selectivity (α)	-	2.46	1.23	-	3.96	2	> 1
Number of theoretical Plate (N)	2483	17,039	22,390	-			> 2000
Height equivalent to theoretical plate (HETP)	0.10068	0.07096	0.04577	-			The smaller the value, the higher the column efficiency

**Table 3:** Robustness assessment of the proposed methods for determination of PHE and DOX.

Parameters		HPLC method						HPTLC method			
		Change in ratio of ethanol (%)		Mobile phase flow rate (mL/min)		Detector wavelength (nm)		Change in ratio of methylene chloride (%)		Saturation time (min)	
		29	31	0.9	1.1	260	261	2	3	25	35
Measured R_t_	PHE	2.77	2.81	2.80	2.77	2.78	2.81	-			
	DOX	5.42	5.39	5.4	5.38	5.4	5.43				
Measured R_f_	PHE	-						0.29	0.32	0.30	0.29
	DOX							0.64	0.63	0.64	0.65
Measured K	PHE	1.80	1.77	1.79	1.81	1.8	1.78	2.22	2.23	2.21	2.23
	DOX	4.42	4.40	4.41	4.39	4.41	4.39	0.56	0.55	0.55	0.57
Measured T	PHE	0.95	0.94	0.96	0.95	0.93	0.94	0.95	0.96	0.94	0.95
	DOX	1.02	1.01	1.02	1	1.01	1.02	1.16	1.15	1.15	1.16

### Assay of dosage form and statistical comparison

By using the suggested techniques, PHE and DOX in their pharmaceutical dosage form were successfully determined. Table 4 displays the mean recoveries and RSD% that were determined. Additionally, the methods’ validity was evaluated using the standard addition technique. PHE and DOX in Poly Hist Forte^®^ tablets were successfully determined using the developed methods. Table 5 illustrates the results of a statistical comparison between the official titrimetric method^14^ and the developed methods, which revealed no statistically significant differences.

**Table 4:** Determination of PHE and DOX in its tablet dosage form and application of standard addition technique using the established methods. ^a^ average of 3 determinations of tablet dosage form.

	PHE						DOX					
	HPLC method			HPTLC method			HPLC method			HPTLC method		
	Taken (µg/mL)	Added (µg/mL)	Recovery %	Taken (µg/band)	Added (µg/band)	Recovery %	Taken (µg/mL)	Added (µg/mL)	Recovery %	Taken (µg/band)	Added (µg/band)	Recovery %
Dosage form (10 mg PHE/10.5 mg DOX)	6.00	3.00	101.66	6.00	3.00	101.54	6.50	3.25	101.57	6.50	3.25	99.49
		6.00	99.01		6.00	100.81		6.50	98.94		6.50	100.56
		12.00	98.69		12.00	100.65		13.00	98.66		13.00	99.49
	Mean^a^ % ± SD		99.78 ± 1.63	Mean^a^ % ± SD		101.04 ± 0.47	Mean^a^ % ± SD		99.72 ± 1.60	Mean^a^ % ± SD		99.84 ± 0.61

**Table 5:** Statistical comparison of the obtained results by applying the developed methods and the official method for the determination of PHE and DOX in their pharmaceutical preparation. ^a^ average of 6 experiments. ^b^ figures between parentheses represent the corresponding tabulated values of t and f at *P* = 0.05.

Value	PHE			DOX		
	HPLC method	HPTLC method	Official method^14^	HPLC method	HPTLC method	Official method^14^
Mean (%)^a^	99.68	99.15	101.29	99.41	99.64	100.35
SD	0.576	0.468	1.595	0.659	1.213	1.135
n	3	3	3	3	3	3
Variance	0.331	0.219	-	0.433	1.47	-
Student’s t-test (2.78) ^b^	1.64	2.23	-	1.24	0.74	-
F value (19.05) ^b^	7.68	11.60	-	2.97	1.14	-

### Greenness assessment

#### Analytical greenness metric (AGREE)

**Fig. 5:**
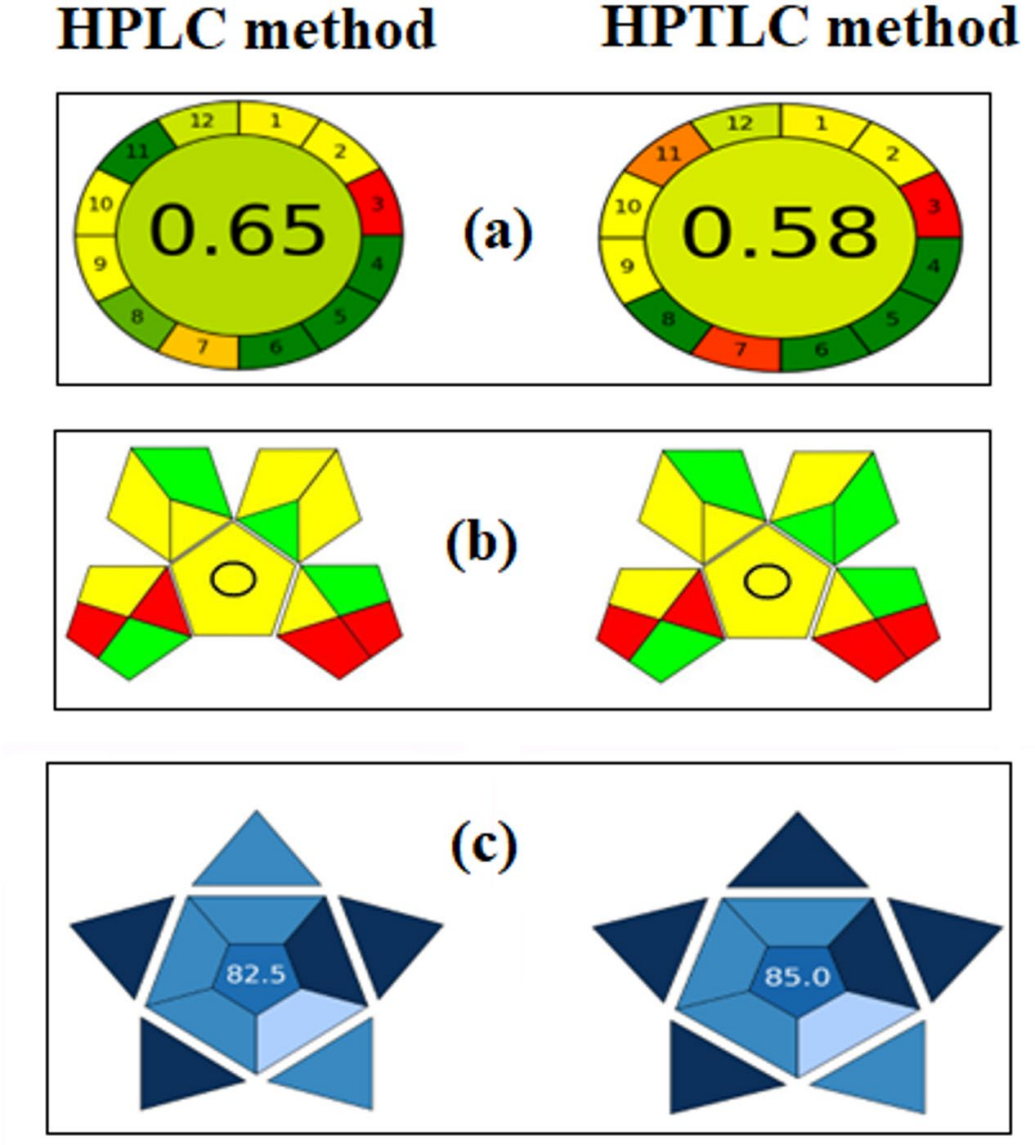
(**a**) AGREE assessment of the green profile, (**b**) GAPI pictograms, (**c**) BAGI tools for the proposed methods.

The 12 input variables, which were calculated for suggested approaches, are displayed as colored pictograms in (Fig. 5a). The AGREE scores of the established methods were 0.65 and 0.58 for HPLC and HPTLC, respectively.

#### Green analytical procedure index (GAPI)

It was necessary to evaluate an analytical method’s environmental impact to ascertain whether it adhered to the principles of green chemistry. The HPLC method received four red, four green, and seven yellow ratings, while the HPTLC method received four red, five green, and six yellow ratings as shown in (Fig. 5b). This profile explains how the authorized technique is easy to use, safe for the environment, and capable of being used for both characterization and measurement without the need for extraction techniques. It also offered a straightforward process for cutting down on waste and the use of dangerous materials.

### Blue applicability grade index (BAGI)

Ten key parameters were computed for both established HPLC and HPTLC approaches, and the results in (Fig. 5c) showed equal scores of 82.5 and 85.5, respectively.

The novelty of this work is concerned with four advantages over the previously published method that is used for the determination of DOX and PHE in ternary mixture. First, the established methods were HPLC and HPTLC for the first time, while the reported one was HPLC only. Second, established methods were applied to the separation of DOX and PHE as a new binary mixture in one tablet in addition to in the presence of DOX DEG, while the reported one was used for the determination of DOX, PHE, and DEX as a ternary mixture in a synthetic mixture, not in real dosage form and without any degradation product. Third, ecofriendly used solvents: the solvents used in established approaches were ethanol and phosphate buffer, which they considered as green solvents due to the results of the AGREE and GAPI scores. While in the reported one, the solvents were methanol, acetonitrile, and phosphate buffer, which are highly toxic solvents for humans’ health and the environment. Fourth, retention time in the developed method was 2.79 min and 5.41 min for PHE and DOX, respectively, while in the reported one it was 5.12 min and 10.41 min for PHE and DOX. Finally, the recommended methods were better than the reported one due to the previous reasons, and they were saving time and solvents as shown in (Table 6).

**Table 6:** A comparison between developed methods for binary mixture in presence of degradation product and reported method for ternary mixture.

Items	Developed methods	Reported method	Comment on comparison
Active ingredients	DOX and PHE	DOX, PHE and DEX	They are two different dosage forms and different combinations as in developed methods was binary mixture while reported method was ternary mixture
Methods	HPLC and HPTLC	HPLC only	The developed methods were better than reported as they were 2 separation methods and for first time ever HPTLC method
Determination	Binary mixture in presence of DOX alkaline degradation product	Ternary mixture only without degradation product	Stability used in developed methods and approved degradation product by LC-MS
Solvents	In HPLC: ethanol and phosphate buffer In HPTLC: Methylene chloride, 30% ammonia, and ethanol	Methanol: Acetonitrile and phosphate buffer	Solvents which used in developed methods were greener than those used in reported one as methanol and acetonitrile were toxic and harmful solvent while solvent in developed methods were ecofriendly as calculated by AGREE and GAPI.
Retention time	PHE: 2.79 min DOX:5.41 DOX DEG: 6.45 min	PHE : 5.12 min DOX : 10.41 min DEX : 2.661 min	The developed method was better than reported one as it was time saving and consumed low amount of solvent while reported was not.
Dosage form	Binary mixture present in one tablet and its name was Poly Hist Forte^®^ tablets	It was a synthetic mixture not real dosage form contain 3 drugs in one tablet	Developed methods were better in this point

## Conclusion

Pharmaceutical research is becoming more interested in greening analytical techniques to reduce negative environmental effects of solvents and improve analyst health. In this study, the first green chromatographic approaches were developed for the simultaneous determination of PHE and DOX in routine pharmaceutical analysis and in the presence of degradation products. The sustainability of the developed methods was measured by applying three approaches such as GAPI, AGREE and BAGI. Given this, the proposed methods may be a useful and safe replacement for routine analysis of the pharmaceutical mixture under investigation, particularly in labs lacking more sophisticated equipment. The statistical comparison between the suggested protocols and the official one revealed no discernible differences in terms of sensitivity, accuracy, and precision in accordance with ICH guidelines.

## Supplementary Information

Below is the link to the electronic supplementary material.

Supplementary Material 1

## Data Availability

The datasets used and/or analyzed during the current study are available from the corresponding author on reasonable request.
