# Supplementary material for: Two sustainable chromatographic approaches for estimation of new combination of phenylephrine hydrochloride and doxylamine succinate in presence of doxylamine oxidative degradation product
Source: Sci Rep. 2025 Oct 23;15:37156. doi: 10.1038/s41598-025-22295-6 (PMC12549921; doi:10.1038/s41598-025-22295-6)
Supplement: Supplementary file 1 — Supplementary Material 1 [file 41598_2025_22295_MOESM1_ESM.docx]

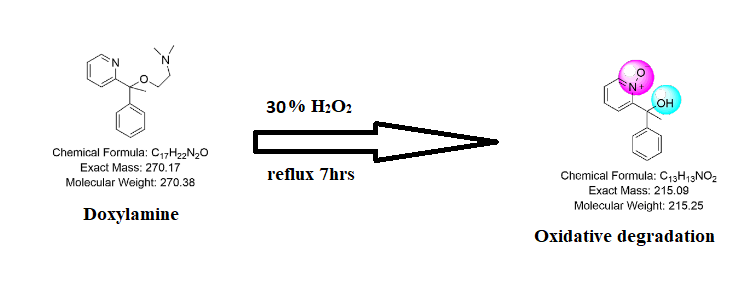


**Figure. S1.** Schematic diagram of the suggested degradation pathway of DOX.


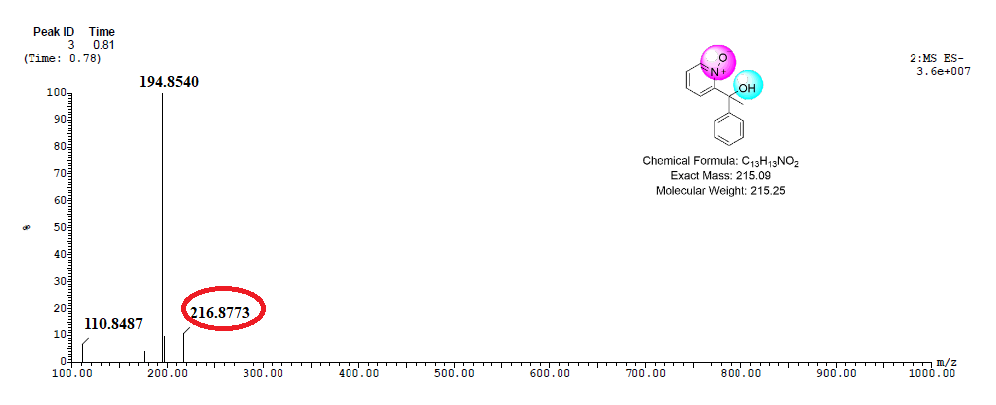


**Figure.S2**: LC-Mass spectra of DOX sample showing M.wt .of degradation product
